# *Petroselinum sativum* (Parsley) extract suppresses oxidative stress and inflammatory responses in human keratinocytes and reduces atopic dermatitis symptoms in mouse skin

**DOI:** 10.3389/fphar.2025.1646822

**Published:** 2025-09-12

**Authors:** Juan Wang, Xiaoqian Wu, Huihao Tang, Zhiwei Liu, Yun Ding, Minyi Feng, Shasha Wang, Jiaqi Zuo, Qi Zhao, Yaozhao Li, Chuntao Zhai, Zhenlin Hu, Xiaolei Ding, Nan Liu

**Affiliations:** ^1^ School of Medicine, Shanghai University, Shanghai, China; ^2^ LB Cosmeceutical Technology Co., Ltd., Shanghai, China; ^3^ Hangzhou Island Xingqing Biotechnology Co., Ltd., Hangzhou, China

**Keywords:** *Petroselinum sativum*, antioxidant, anti-inflammation, keratinocytes, atopicdermatitis

## Abstract

*Petroselinum crispum* (Mill.) Fuss (parsley), a traditional botanical drug used for treating skin conditions including atopic dermatitis (AD), has unclear effects on epidermal keratinocytes. This study investigated the antioxidant and anti-inflammatory properties of parsley extracts in human keratinocytes and evaluated their therapeutic potential in an experimental AD model. The aqueous, ethanolic, and hydro-ethanolic (HE) extracts of parsley were evaluated for total polyphenol and flavonoid metabolites (TPC, TFC) and antioxidant activity using DPPH and FRAP assays. *In vitro*, HaCaT cells were treated with tert-butyl hydroperoxide (t-BHP) and TNF-α/IFN-γ to induce oxidative stress and inflammation. Therapeutic efficacy was further evaluated in 2,4-dinitrofluorobenzene (DNFB)-induced AD-like mouse model. The results showed that HE extracts of parsley (HEP) contained the highest TPC and TFC and exhibited the strongest antioxidant activity, significantly improving cell viability and reducing ROS levels in t-BHP-treated cells. Mechanistically, HEP alleviated oxidative stress by activating Nrf2 pathway and enhancing the expression of antioxidant enzymes, such as superoxide dismutase (SOD) and catalase (CAT). In addition, HEP suppressed inflammatory cytokines IL-33, IL-6, and IL-8 expression by inhibiting JAK1/STAT1 and NF-κB signaling, and simultaneously increased the expression of skin barrier proteins, including filaggrin and claudin-1 in TNF-α/IFN-γ-stimulated HaCaT cells. Moreover, HEP application could alleviate AD-like symptoms in DNFB-induced mouse model, including reduced skin hyperplasia and decreased immune cells infiltration. These findings suggest that HEP modulates oxidative stress and inflammation through multiple signaling pathways, offering promising natural therapeutic agent for AD management.

## 1 Introduction

Keratinocytes, the primary cell type in the epidermis, are central to skin function and integrity. They not only form the first line to defense against external threats, such as pathogens and UV radiation, but also play a crucial role in immune surveillance (Watt, 2014). Through the activation of innate immune receptors, keratinocytes initiate a cascade of immune responses, producing cytokines, chemokines, and antimicrobial peptides that protect the skin from microbial invasion and regulate local inflammation (Chieosilapatham et al., 2021; Albanesi and Pastore, 2010). Dysregulation of keratinocyte functions compromises the skin barrier, a hallmark of inflammatory skin disorders like atopic dermatitis (AD). Additionally, AD is characterized by excessive inflammation and heightened susceptibility to infections, posing significant therapeutic challenges (Yang et al., 2020; Morizane et al., 2023; Schuler et al., 2023).

Oxidative stress and chronic inflammation are key contributors to the onset and progression of AD (Ji and Li, 2016; Raimondo, Serio, and Lembo, 2023; Bertino et al., 2020). The imbalance between reactive oxygen species (ROS) and antioxidant defenses in the skin exacerbates tissue damage, while persistent inflammation further impairs barrier function and accelerates disease progression (Wullaert, Bonnet, and Pasparakis, 2011; Bertino et al., 2020). Consequently, strategies that modulate oxidative stress and inflammation hold significant therapeutic potential for managing AD and other related skin conditions (De Simoni et al., 2024; Li et al., 2021).

*Petroselinum crispum* (Mill.) Fuss (parsley), a widely used botanical drug, has recently attracted increasing attention for its therapeutic effects on skin health, particularly due to its antioxidant and anti-inflammatory properties. Containing bioactive metabolites, such as polyphenols and flavonoids, parsley has demonstrated a wide range of biological activities, including modulation of oxidative stress and inhibition of inflammatory pathways (Bahramsoltani et al., 2024; Farzaei et al., 2013; Mahmood, Hussain, and Malik, 2014). The application of parsley-derived formulations has recently been investigated for dermatological benefits (Slighoua et al., 2023; Khosravan et al., 2017; Ganea et al., 2024). For example, topical application of HE and polyphenol extracts of parsley (10% (w/w)) has been shown to suppress wound infections and enhance burn healing. Despite these benefits, the effects and molecular mechanisms through which parsley extracts influence keratinocytes remain unclear.

In this study, we investigated the effects of the hydro-ethanolic extract of parsley (HEP) on oxidative stress, inflammatory responses, and skin barrier function in human HaCaT keratinocytes and an AD mouse model. Our findings indicate that HEP is rich in bioactive polyphenolic and flavonoid metabolites, which play a crucial role in modulating oxidative stress and inflammatory responses *in vitro* and *in vivo*. Mechanistically, HEP modulates the activities of Nrf2, JAK/STAT and NF-κB signaling pathway, which play crucial roles in cellular antioxidant responses, inflammation, and immune regulation. A deep understanding of the molecular mechanisms underlying parsley extract could provide valuable insights into its therapeutic potential for treating inflammatory skin diseases such as AD.

## 2 Materials and methods

### 2.1 Plant material and preparation of extracts

Parsley root of *P. crispum* (Mill.) Fuss (syn. *Petroselinum sativum* Hoffm., Apiaceae) was purchased from a local herbal market in Shanghai, China, where the plants were traditionally cultivated on an organically composed natural substrate. The plant materials were taxonomically authenticated by Prof. Guixin Chou, Institute of Chinese Materia Medica, Shanghai University of Traditional Chinese Medicine, Shanghai, China.

After air-drying, the roots were ground into a fine powder to obtain the crude extract. To obtain the aqueous extract of parsley (AP), each plant powder (100 g) was macerated in 900 mL of distilled water for 1 h at 95 °C. The HEP was obtained by reflux extracting plant powder using a mixture of ethanol and distilled water (50:50 V/V) in a ratio of 100 g powder to 1,000 mL of hydro-ethanolic solvent for 1 h at 90 °C. Ethanolic extracts of parsley (EP) were obtained by reflux extraction of 100 g plant powder using 1,000 mL of 95% alcohol for 1 h at 80 °C. In all cases, the extracts were filtered (Whatman No. 1), concentrated by rotary evaporation under reduced pressure, and subsequently freeze-dried to constant weight. The extraction yields were as follows: 16.35 g (AP), 12.35 g (HEP), and 7.90 g (EP), corresponding to drug–extract ratios (DER) of approximately 6.12:1, 8.10:1, and 12.66:1, respectively. Dried extracts were stored in airtight containers at 4 °C until use.

Extracts were chemically characterized to ensure batch-to-batch consistency. Flavonoid constituents of HEP extract were initially profiled using Liquid Chromatography-Mass Spectrometry (LC-MS) as previously described (Obmann et al., 2011), enabling the identification of key bioactive compounds. Quantitative analysis of the principal marker flavonoids, isorhamnetin, quercetin, and luteolin, was subsequently carried out using High-Performance Liquid Chromatography (HPLC), with reference standards for each compound. Details of the extraction procedure, chromatographic conditions, and representative results were provided in the Supplementary Methods and Supplementary Tables S1 and S2. The phytochemical evaluation and documentation of the extract followed the GA best-practice and ConPhyMP (Consensus on the Phytochemical Characterization of Medicinal Plant Extracts) reporting guidelines for botanical products (Heinrich et al., 2022), and the corresponding completed GA checklist tables are included as Supplementary Material.

### 2.2 Reagents

Recombinant human TNF-α and IFN-γ were from Peprotech (Rocky Hill). Tert-Butyl hydroperoxide (t-BHP) and other chemicals were purchased from Sigma-Aldrich. Primary antibodies for Nrf2 (cat. no. 12721), NF-κB P65 (cat. no. 8242), IκBα (cat. no. 4814), p-JAK1 (cat. no. 74129), JAK1 (cat. no. 3344), p-STAT1 (cat. no. 9167), STAT1 (cat. no. 14994), and β-actin (cat. no. 4970) were obtained from Cell Signaling Technology. The primary antibodies against anti-Lamin B (cat. no. ab16048) were purchased from Abcam. Horseradish peroxidase-conjugated secondary antibodies were purchased from Jackson ImmunoResearch Laboratories.

### 2.3 Analysis of total polyphenol and flavonoid metabolites in parsley extracts

Total polyphenol content (TPC) was determined using the Folin-Ciocalteu method (Pyeon et al., 2021). Briefly, the extract was dissolved in deionized water. Dissolved extract solution (1 mL) was mixed with 9 mL of distilled deionized water (dd H_2_O) and treated with 1 mL of Folin-Ciocalteu reagent (Sigma-Aldrich). After reacting at room temperature for 5 min, this solution was mixed with 10 mL of 7% sodium carbonate (Na_2_CO_3_) and 4 mL of dd H_2_O. The mixture stood at room temperature for 90 min, and the absorbance was measured at 750 nm by a fluorescence microplate reader (BioTek Instruments). Gallic acid (GA, Sigma-Aldrich) was used as a standard. The data were expressed as mg GA equivalents (GAE)/g of lyophilized extract powder.

Total flavonoid content (TFC) was determined by the aluminum chloride (AlCl_3_) colorimetric assay (Pyeon et al., 2021). The extract was dissolved in deionized water. Dissolved extract solution (1 mL) was mixed with 4 mL of dd H_2_O and treated with 0.3 mL of 5% sodium nitrate (NaNO_2_). After 6 min at room temperature, 0.3 mL of 5% AlCl_3_, 2 mL of 1 M sodium hydroxide (NaOH), and 2.4 mL dd H_2_O were added. Additionally, the absorbance was then measured at 510 nm by a fluorescence microplate reader. Rutin (Sigma-Aldrich) was used as a standard. The data were expressed as mg rutin equivalents (CE)/g lyophilized extract powder.

### 2.4 Assessment of *in vitro* antioxidant activity of parsley extracts

Antioxidant activity of the extracts was evaluated using the DPPH radical scavenging assay and Ferric Reducing Antioxidant Power (FRAP) assay.

#### 2.4.1 DPPH radical scavenging assay

The assay was performed according to the method described previously (Gulcin, 2020) with some modifications. Briefly, 100 μL of the extract and 100 μL of the DPPH solution (0.2 mM) were mixed in individual wells of a 96-well microplate. After 30 min of incubation in the dark, the optical density (OD) at a wavelength of 517 nm (OD_517_) was measured using a microplate reader (BioTek Epoch). All the experiments were performed in triplicate. Trolox was used as the standard antioxidant. To calculate DPPH radical scavenging rate (%), the following formula was used:

$$\text{DPPH radical scavenging rate }\left(\%\right)=\left(A_{\text{blank}}\;‐\;A_{\text{sample}}/A_{\text{blank}}\right)\times 100$$

$$A_{\text{blank}}:{\text{OD}}_{517}\text{ of DPPH solution in the absence of the extract or Trolox}$$

$$A_{\text{sample}}:{\text{OD}}_{517}\text{ of DPPH solution containing the extract or Trolox}$$

The DPPH radical scavenging rate (%) was plotted against different concentrations of the tested substance (parsley extracts or Trolox). The IC_50_ value, representing the concentration required to inhibit 50% of the DPPH radicals, was calculated from the linear regression of the scavenging rate plotted against extract concentrations.

#### 2.4.2 FRAP assay

FRAP assay was carried out in a 96-well plate as described previously (Gulcin, 2020), with modifications. Briefly, the FRAP reagent was freshly prepared by mixing acetate buffer (300 mM), TPTZ (10 mM in HCl, 40 mM), and FeCl_3_ (20 mM in distilled water) in a ratio of 10:1:1 (v/v/v), then incubated at 37 °C for 10 min. For analysis, 150 μL of the FRAP solution was mixed with 30 μL of the samples and incubated at room temperature for 30 min. The optical density at a wavelength of 593 nm (OD_593_) was measured using a microplate reader (BioTek Epoch). The results were expressed as EC_50_, calculated from the standard curve, representing the concentration required for 50% of the maximum antioxidant activity.

### 2.5 Cell culture

HaCaT cells were purchased from The Cell Bank of Chinese Academy of Sciences. Cells were cultured in Dulbecco’s modified Eagle medium (DMEM) (Sigma-Aldrich), supplemented with 10% (v/v) fetal bovine serum (FBS) (Gibco) and 1% penicillin/streptomycin (Gibco) at 37 °C and in a humidified 5% CO_2_ incubator.

### 2.6 Cell viability assay

Cell viability was measured with a Cell Counting Kit-8 (CCK-8) assay (APExBio) according to the manufacturer’s instructions. The HEP solution was prepared by dissolving lyophilized HEP in DMSO to generate a 50 mg/mL stock solution. Prior to cell treatment, this DMSO stock was diluted with culture medium to achieve final treatment concentrations. HaCaT cells were seeded in 96-well plates at a density of 1 × 10^4^ cells per well and incubated for 24 h. Cells were treated for 6 h with different concentrations of the hydro-ethanolic extract of parsley and then treated with 0.4 mM t-BHP for 2 h. After that, the cells were cultured with fresh medium supplemented with the same concentrations of HEP for another 24 h. 15 μL of CCK-8 reagent (5 mg/mL) was then added to cells and incubated for 2 h at 37 °C. The optical density at a wavelength of 450 nm (OD_450_) was measured using a microplate reader (BioTek Epoch). Each measurement was performed in triplicate. Cell viability was expressed relative to that of the vehicle control group.

### 2.7 Estimation of intracellular ROS level

Intracellular ROS levels in t-BHP-stimulated HaCaT cells were estimated by DCFH-DA fluorescence staining method. HaCaT cells were seeded in 6-well plates at a density of 1 × 10^5^ cells/well for 24 h. Cells were pretreated with different concentrations of the HEP for 6 h and then stimulated with t-BHP for 2 h. Subsequently, cells were further incubated with 10 μM DCFH-DA (diluted in fresh DMEM) for 30 min at 37 °C. DCFH-DA labeled intracellular ROS were observed under an Olympus fluorescent microscope. Fluorescence intensity of the DCFH-stained cells for each condition was quantified by Olympus Softimage solution software (Olympus Imaging America), and data were represented as fold change relative to untreated control values.

### 2.8 Western blotting

Total protein was extracted from HaCaT cells using cell lysis buffer (Beyotime Biotechnology, Shanghai, China). Nuclear and cytoplasmic protein fractions were extracted from HaCaT cells using an extraction reagent kit (Beyotime Biotechnology, Shanghai, China), according to the manufacturer’s instructions. The protein concentration was determined by a modified BCA protein assay (Beyotime Biotechnology, Shanghai, China). About 20 μg protein was subjected to electrophoresis on an SDS–polyacrylamide gel, and transferred onto a polyvinylidene difluoride (PVDF) membrane. The membranes were incubated with a blocking solution (5% BSA dissolved in Tris-buffered saline with 0.05% TWEEN-20 (TBST) for 30 min at room temperature and then incubated overnight with a primary antibody (dilution, 1:1,000 in TBST) at 4 °C. After washing thrice with TBST, the blots were incubated with a horseradish peroxidase-conjugated secondary antibody (West Grove) (dilution, 1:2,000) for 2 h at room temperature and washed again thrice with TBST. The blots were visualized using a SuperPico ECL Chemiluminescence Kit (Nanjing Vazyme Biotech). Densitometry was performed using the ImageJ software.

### 2.9 RNA extraction and real time quantitative polymerase chain reaction (qPCR)

Total RNA was extracted from HaCaT cells using an RNA isolation kit (Yeasen Biotechnology Co., Ltd., Shanghai, China) according to the manufacturer’s instructions. For each sample, 1 µg total RNA was reverse transcribed into cDNA using PrimeScript RT reagent Kit (Accurate Biotechnology Co., Ltd., Hunan, China). qPCR was performed on the quantum Studio 6 real-time PCR system (Thermo Fisher Scientific, Inc., Waltham, MA, United States) using SYBR Green Master Mix (Accurate Biotechnology Co., Ltd., Hunan, China). The sequences of used primers are presented in Table 1. The relative messenger RNA (mRNA) level of each target gene was quantified using the 2^−ΔΔCT^ method and normalized to that of β-actin.

**TABLE 1:** Primer sequences used for qPCR.

Gene	Forward primer (5′-3′)	Reverse primer (5′-3′)
*SOD*	GGT​GGG​CCA​AAG​GAT​GAA​GAG	CCA​CAA​GCC​AAA​CGA​CTT​CC
*CAT*	ATT​CTG​GAG​AAG​TGC​GGA​GA	CGG​CAA​TGT​TCT​CAC​ACA​GA
*FLG*	TGA​AGC​CTA​TGA​CAC​CAC​TGA	TCC​CCT​ACG​CTT​TCT​TGT​CCT
*Claudin-1*	CCG​TGC​CTT​GAT​GGT​GGT​TGG	CAT​CTT​CTG​CAC​CTC​ATC​GTC​TTC​C
*IL-33*	GGA​AGA​ACA​CAG​CAA​GCA​AAG​CC	GGC​CAG​AGC​GGA​GCT​TCA​TAA​AG
*IL-6*	ACT​CAC​CTC​TTC​AGA​ACG​AAT​TG	CCA​TCT​TTG​GAA​GGT​TCA​GGT​TG
*IL-8*	AGA​GAG​CTC​TGT​CTG​GAC​CC	TTC​TCA​GCC​CTC​TTC​AAA​AAC​T
*β-actin*	GTA​CGC​CAA​CAC​AGT​GCT​G	CGT​CAT​ACT​CCT​GCT​TGC​TG

### 2.10 Animals and induction of AD-like symptoms

All *in vivo* studies were approved by Institutional Animal Care and Use Committee of Shanghai University (project approval number ESSHU2024-003). After 1 week of acclimation, mice were randomly divided into the following four groups: (1) Control (Ctrl): Untreated mice receiving vehicle only; (2) 2,4-dinitrofluorobenzene (DNFB): Mice treated with DNFB to induce AD-like dermatitis; (3) DNFB+ dexamethasone (DEX): DNFB-treated mice receiving DEX (positive control); (4) DNFB+HEP: DNFB-treated mice receiving HEP. The hapten-induced AD model was established using a two-phase sensitization protocol (Yang et al., 2023). On day 0, mice were sensitized by topical application of 25 μL of 1% DNFB (Sigma, St. Louis, MO, United States) dissolved in acetone/olive oil (4:1 v/v) to the shaved abdominal skin (Figure 8A). On day 5, mice received challenge applications of 5 μL of 0.4% DNFB solution to both inner and outer ear surfaces to elicit localized inflammatory responses (Ding et al., 2020). Given the rapid anti-inflammatory action of DEX during peak inflammation, HEP was administered prophylactically on Day 3 post-induction (1% DNFB), whereas DEX intervention commenced at the inflammatory peak on Day 5. Briefly, HEP group: Mice received daily pretreatment with HEP (50 mg/kg) for two consecutive days prior to 0.4% DNFB challenge. DEX group: Mice received daily subcutaneous injections of DEX (2 mg/kg/day) from day 5. All treatment groups except controls received a final 0.4% DNFB application on day 5 to maintain inflammatory stimulation. Disease severity was evaluated using a standardized scoring system applied by blinded observers (Shi et al., 2019). The severities of erythema (hemorrhage), edema, erosion, and dryness in individual mice were scored as 0 (absent), 1 (mild), 2 (moderate), and 3 (severe) in a blinded manner. Ear thickness was measured at 0, 8, 24, and 48 h after DEX administration with a digital caliper. Ear thickness was calculated by four groups compared with Ctrl group. The tissue samples were collected on day 7, and used for subsequent experiments.

### 2.11 Histological analysis and immunohistochemistry staining

Mouse ear skin tissues were collected and fixed with 4% paraformaldehyde (PFA) in PBS overnight before paraffin embedding, and sectioned. Tissue sections (4 μm) were stained with hematoxylin and eosin (H&E) and toluidine blue (TB, Biosharp). Ear epidermal thickness was taken using ImageJ software.

The expression of CD3 and CD68 was detected using immunohistochemistry staining as described previously (Tang et al., 2024; Liu et al., 2024). The tissues sections underwent heat-induced epitope retrieval at 95 °C for 20 min. After blocking with 3% BSA, the slides were stained with primary antibodies: CD3 (Abcam: ab16669), CD68 (Cell Signaling Technology: #97778) overnight at 4 °C. Subsequently, sections were incubated with HRP-conjugated secondary antibody (Goat anti-rabbit IgG) for 1 h at RT. DAB substrate kit was used to detect the signal. Images were taken using the Olympus Microscope IX71 (Olympus, Tokyo, Japan). Five visions were counted per tissue section used for measurement. The mean intensity and cell quantity of skin tissue was quantified from photographs using ImageJ software with the IHC Toolbox.

### 2.12 Statistical analysis

All data were expressed as a mean ± standard deviation (SD) from three independent experiments. Comparisons among groups were carried out using one-way analysis of variance followed by Dunnett’s post hoc test using GraphPad Prism 6.0 software (Graphpad Software). Statistical significance was set at *p* < 0.05.

## 3 Results

### 3.1 Parsley extracts exhibit high TPC and TFC levels

The analysis of TPC and TFC revealed significant variations among AP, EP and HEP. Notably, HEP demonstrated the highest concentration of both polyphenols (43.87 ± 0.09 mg GA/g extract) and flavonoids (57.76 ± 0.30 mg rutin/g extract) (Table 2), indicating superior extraction efficiency of bioactive constituents. LC-MS profiling was employed to identify the major flavonoid constituents of HEP extract (Supplementary Methods). HPLC was used to quantify key marker compounds, including isorhamnetin, quercetin, and luteolin. The results from HPLC corroborated the LC-MS findings, confirming comparable levels of these flavonoids across analytical methods (Supplementary Tables S1 and S2). These findings suggest that the hydro-ethanolic extraction method is more effective in extracting TPC and TFC, the key bioactive metabolites from parsley.

**TABLE 2:** Total polyphenol and flavonoid contents in parsley extracts.

Parsley extracts	Total polyphenols (mg GA/g extract)	Total flavonoids (mg rutin/g extract)
HEP	43.87 ± 0.09	57.76 ± 0.30
AP	27.60 ± 0.16	25.62 ± 0.22
EP	26.28 ± 0.37	36.78 ± 0.08

### 3.2 *In vitro* antioxidant activity of parsley extracts

**FIGURE 1:**
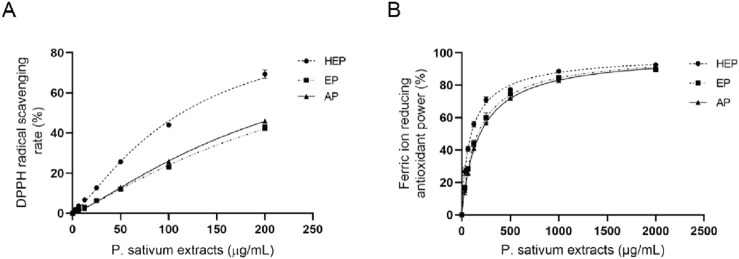
HEP displays superior antioxidant potency. Antioxidant activity of different parsley extracts (HEP, EP, and AP) using **(A)** DPPH and **(B)** FRAP assays.

The antioxidant capacity of parsley extracts was evaluated using DPPH and FRAP assays. Among the extracts, HEP showed the highest DPPH scavenging activity with an IC_50_ of 113.467 μg/mL, while AP and EP had higher IC_50_ (221.800 μg/mL and 239.067 μg/mL, respectively) (Figure 1A; Table 3), indicating a decreased potency in scavenging DPPH radicals for the latter two extracts. Similarly, the FRAP assay demonstrated that HEP had the greatest ferric reducing antioxidant power with an EC_50_ of 96.42 μg/mL, compared to AP (186.50 μg/mL) and EP (164.97 μg/mL) (Figure 1B; Table 3). These findings highlight the superior antioxidant potency of HEP, which correlates with its higher TPC and TFC content in parsley extracts.

**TABLE 3:** Antioxidant activity of parsley extracts using DPPH and FRAP assays.

Parsley extracts	DPPH IC_50_ (μg/mL)	DPPH IC_50_ (μg/mL)
HEP	113.47 ± 6.40	96.42 ± 9.92
AP	239.07 ± 8.41	164.97 ± 17.26
EP	227.1 ± 5.77	186.50 ± 10.26

### 3.3 Protective effect of HEP on oxidative damage in keratinocytes

**FIGURE 2:**
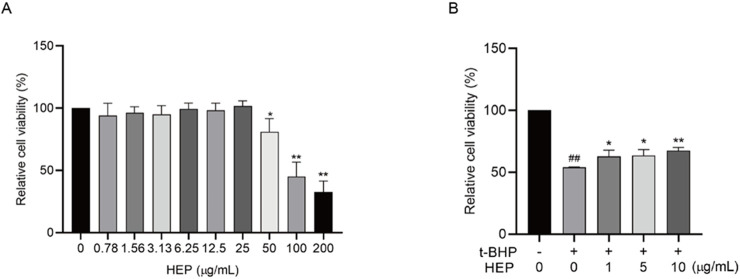
The protective effect of HEP on t-BHP-induced oxidative damage in HaCaT cells. **(A)** HaCaT cells were treated with indicated concentrations of HEP for 24 h, and the cell viability was assayed with CCK8 assay. **(B)** HaCaT cells were treated with HEP for 6 h at indicated concentrations and then treated with 0.4 mM t-BHP for 2 h. After that, the cells were cultured with fresh medium supplemented with the same concentrations of HEP for another 24 h. Cell viability was then measured using CCK-8 assay. The data are presented as mean ± SD of three independent experiments. *p < 0.05, **p < 0.01 vs t-BHP alone control; ^##^ *p* < 0.01 vs non-treatment control.

Given the superior potential of HEP, our following experiments mainly concentrated on investigating its activities. The possible cytotoxic effects of HEP on HaCaT cells were first evaluated via CCK-8 assay. The results revealed that HEP at concentrations of 25 μg/mL or lower did not exhibit any effect on the cell viability over a 24-h period (Figure 2A), excluding the cytotoxicity on HaCaT cells. Therefore, further experiments were performed at concentrations of 1, 5, and 10 µg/mL of HEP. t-BHP is an organic peroxide that can be metabolized by cytochrome P450 in cells to generate peroxyl and alkoxyl radicals or detoxified to tert-butanol, both of which can inflict oxidative damage to cells. t-BHP is a commonly used trigger to induce oxidative stress *in vitro* and *in vivo* (Taffe et al., 1987; Bellomo, Thor, and Orrenius, 1984; Kučera et al., 2014; Pinho, Oliveira, and Cunha-Oliveira, 2025). To assess the protective effect of HEP on t-BHP-induced oxidative damage, HaCaT cells were pretreated with HEP (1, 5, and 10 μg/mL) for 6 h and then exposed to 0.4 mM t-BHP. The CCK-8 assay demonstrated that t-BHP stimulation inhibited cell viability, and HEP pretreatment alleviated its inhibition (Figure 2B), suggesting that HEP protected cells from t-BHP-induced oxidative damage.

### 3.4 Effect of HEP on intracellular ROS levels in t-BHP-stimulated HaCaT cells

**FIGURE 3:**
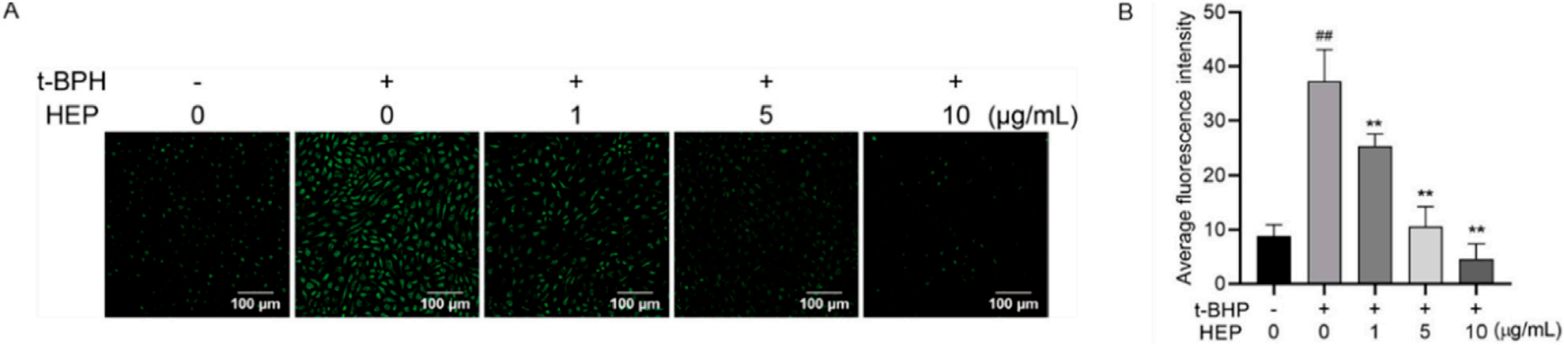
Effect of HEP on intracellular ROS levels in t-BHP-stimulated HaCaT cells. HaCaT cells were pretreated with HEP (0, 1, 5, or 10 μg/mL) for 6 h followed by stimulation with t-BHP for 2 h. **(A)** The accumulation of t-BHP-induced ROS was measured using fluorescence microscopy after DCFH-DA staining (×200 magnification). **(B)** Fluorescence intensity of the DCFH-stained cells for each condition was quantified by Olympus Softimage solution software. The data are presented as mean ± SD of three independent experiments. ^**^ *p* < 0.01 vs single t-BHP treatment group; ^##^ *p* < 0.01 vs non-treatment control.

Excessive ROS contributes to the skin inflammation and disruption of skin barrier (Woodby et al., 2020; Sies and Jones, 2020). Since HEP alleviated the t-BHP-induced oxidative damage, we therefore tested whether HEP alleviated the t-BHP-mediated ROS production. DCFH-DA is a fluorescent indicator. Upon oxidation by ROS, it converts into DCF and emits green fluorescence. Hence, fluorescence intensity in cells can be used to indicate the ROS level. HaCaT cells were pretreated with indicated concentrations of HEP (1–10 μg/mL), following with t-BHP treatment. Compared to the untreated control cells, t-BHP stimulation alone triggered a remarkable increase in fluorescence intensity. However, this effect was dramatically alleviated by HEP-pretreatment in a concentration-dependent manner (Figures 3A,B), indicative of the decreased ROS level. Hence, HEP exhibited antioxidant activity in t-BHP stimulated HaCaT cells.

### 3.5 HEP activates the Nrf2 signaling pathway and upregulates antioxidant genes in stimulated HaCaT cells

**FIGURE 4:**
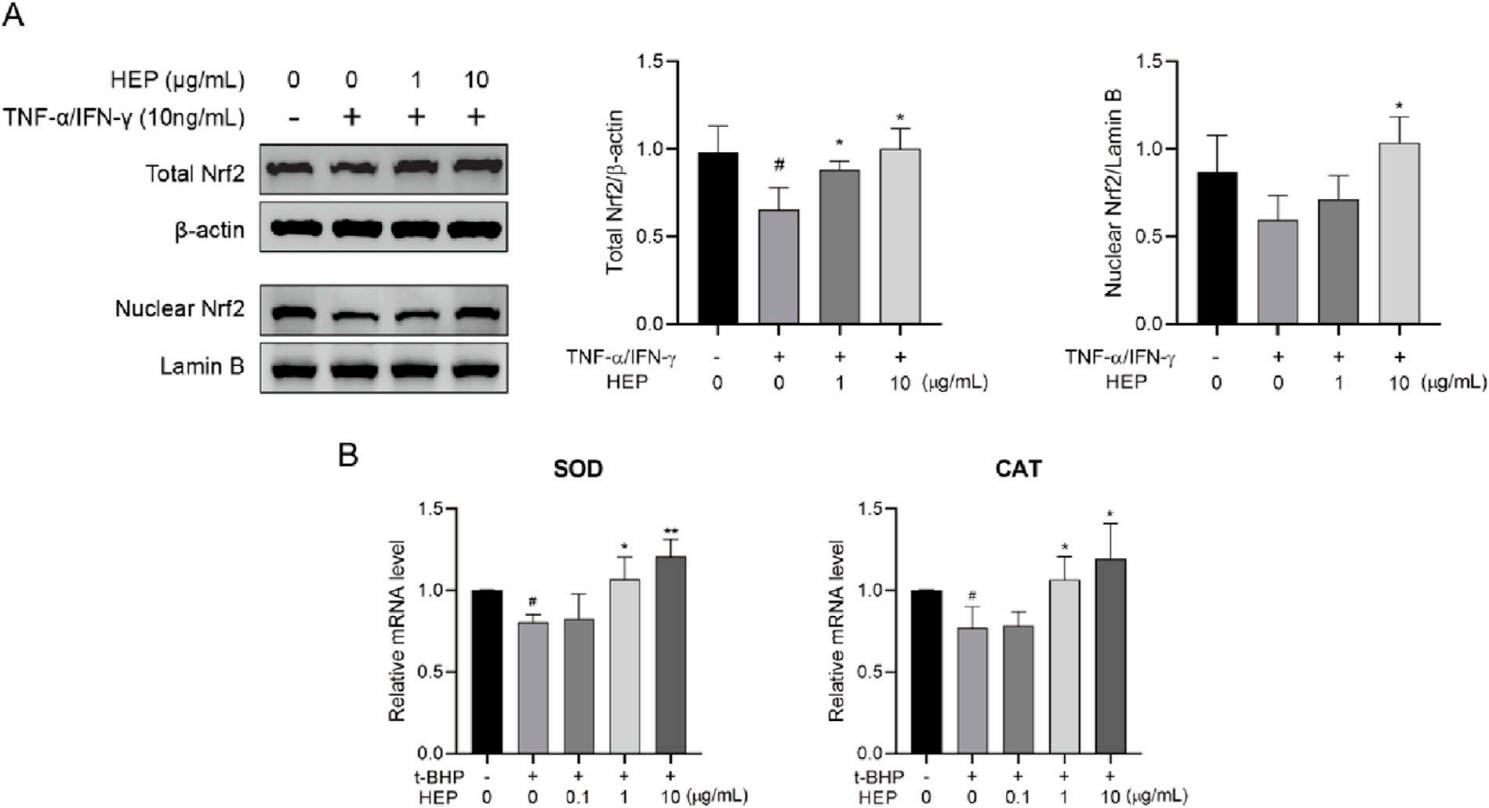
HEP activates Nrf2 signaling pathway in t-BHP-stimulated HaCaT cells. HaCaT cells were treated with the indicated amounts of HEP for 6 h, then stimulated with 0.4 mM of t-BHP for 2 h. **(A)** Nuclear Nrf2 levels and total levels of Nrf2 were assessed by western blotting and quantified by densitometric analysis. **(B)** The mRNA levels of SOD and CAT were measured with qPCR. Data are presented as the mean and quantified by densitometric anal. ^*^ *p* < 0.05, ^**^ *p* < 0.01 vs t-BHP alone control; ^##^ *p* < 0.01 vs non-treatment control.

Nrf2 signaling pathway has an important role in the regulation of oxidative responses (Liu et al., 2022). Therefore, we wondered whether HEP exerted antioxidant activity *via* activating Nrf2. Nuclear-translocated Nrf2, the activated form, was investigated using Western blotting. As shown in Figure 4A, HEP treatment prior to t-BHP stimulation increased nuclear and total Nrf2 protein levels compared to the t-BHP-only group, indicating that HEP treatment resulted in Nrf2 accumulation and nuclear translocation (Figure 4A).

Nrf2 in the nucleus promotes the transcription of multiple antioxidant genes, including superoxide dismutase (SOD) and catalase (CAT). To consolidate the transcription activity, we analyzed the expression of its target genes *via* qPCR. HEP treatment increased mRNA expression levels of SOD and CAT in t-BHP-stimulated HaCaT cells (Figure 4B). These findings support the notion that HEP activates Nrf2 signaling pathway.

### 3.6 HEP suppresses pro-inflammatory cytokine expression in TNF-α/IFN-γ-stimulated HaCaT cells

**FIGURE 5:**
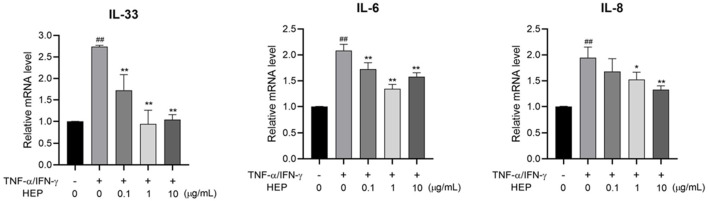
HEP suppresses pro-inflammatory cytokine expression in TNF-α/IFN-γ-stimulated HaCaT cells. HaCaT cells were pre-treated with the indicated amount of HEP for 6 h, then treated with TNF-α/IFN-γ (20 ng/mL) for 24 h. The mRNA expression levels of IL-33, IL-1β, IL-6, and IL-8 were then measured by qPCR. Data are presented as the mean ± SD of three independent experiments. ^##^ *p* < 0.01 vs untreated controls; ^*^ *p* < 0.05, ^**^ *p* < 0.01 vs TNF-α/IFN-γ only control.

To evaluate the anti-inflammatory activity of HEP, the inhibitory effect of HEP on the expression of TNF-α/IFN-γ-stimulated pro-inflammatory cytokines in HaCaT cells were assessed using qPCR. As shown in Figure 5, TNF-α/IFN-γ stimulation significantly increased the mRNA expression levels of IL-33, IL-6, and IL-8 in HaCaT cells, compared to the untreated control group, whereas HEP treatment markedly suppressed the mRNA expression levels of these proinflammatory cytokines in TNF-α/IFN-γ-stimulated HaCaT cells. These findings indicate that HEP possesses anti-inflammatory property.

### 3.7 HEP inhibits JAK/STAT and NF-κB signaling pathway activation in HaCaT cell

**FIGURE 6:**
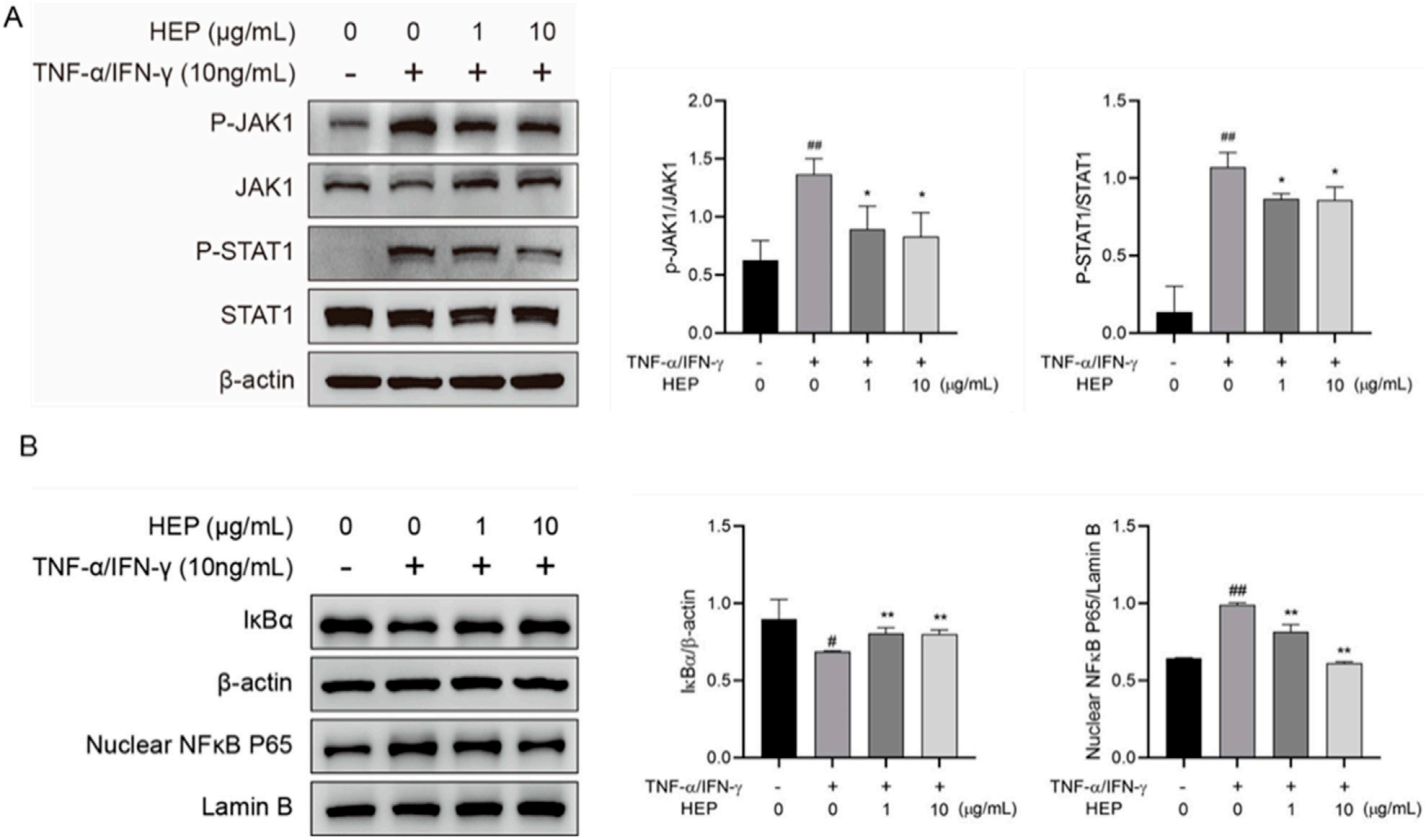
HEP inhibits JAK/STAT and NF-κB signaling pathway activation in HaCaT cell. HaCaT cells were treated with the indicated amount of HEP for 6 h, then stimulated with TNF-α/IFN-γ for 30 min. **(A)** The protein levels of total and phosphorylated JAK1 and STAT1 in the cell lysate were determined by western blotting and quantified by densitometric analysis. **(B)** The protein levels of IκBα and nuclear NF-κB p65 were determined by western blotting and quantified by densitometric analysis. β-actin and Lamin B1 were used as protein control for cytosol and nuclear fractions, respectively. Data are presented as mean ± SD from three independent experiments. ^##^ *p* < 0.01 vs untreated controls; * *p* < 0.05, ** *p* < 0.01 vs TNF-α/IFN-γ only control.

JAK/STAT and NF-κB signaling pathways contribute to TNF-α/IFN-γ-mediated inflammatory responses. Given that HEP inhibited the expression of TNF-α/IFN-γ-induced inflammatory cytokines, we examined its effect on JAK/STAT and NF-κB pathways. Western blot analysis revealed that stimulation of HaCaT cells with TNF-α/IFN-γ markedly induced the phosphorylation of JAK1 and STAT1, and this effect was inhibited by HEP pretreatment (Figure 6A). In addition, TNF-α/IFN-γ stimulation induced degradation of the inhibitor of NF-κB (IκBα) and nuclear translocation of NF-κB subunit p65 (NF-κB p65), whereas HEP treatment inhibited the TNF-α/IFN-γ-induced degradation of IκBα and also prevented the nuclear localization of NF-κB p65 (Figure 6B). These results collectively revealed that HEP inhibited the activation of JAK/STAT and NF-κB signaling pathways in TNF-α/IFN-γ-stimulated HaCaT cells, thereby accounting for its suppressive impact on inflammatory reactions.

### 3.8 HEP increases skin barrier regulator expression in TNF-α/IFN-γ-stimulated HaCaT cells

**FIGURE 7:**
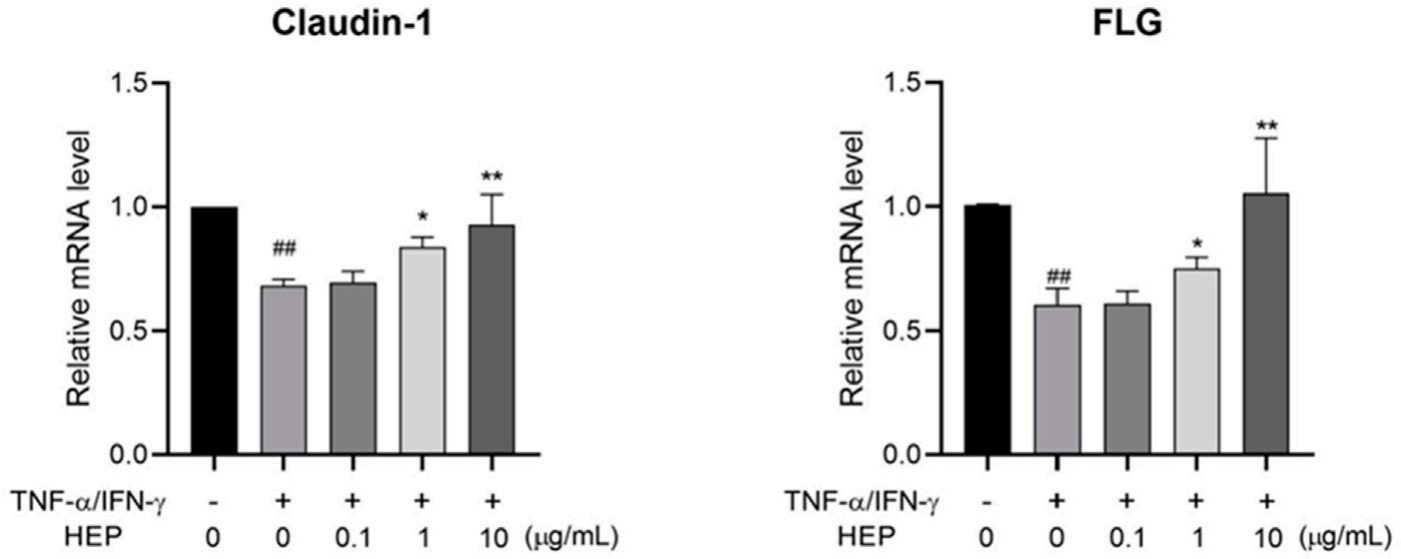
HEP increases skin barrier regulator expression in TNF-α/IFN-γ-stimulated HaCaT cells. HaCaT cells were pre-treated with the indicated amount of HEP for 6 h, then treated with TNF-α/IFN-γ (20 ng/mL each) for 24 h. The mRNA levels of *Claudin-1* and *FLG* were then measured by qPCR. Data are presented as the mean ± SD from three independent experiments. ^##^ *p* < 0.01 vs untreated controls; *p < 0.05, **p < 0.01 vs TNF-α/IFN-γ only control.

To determine the protective potential of HEP on inflammation-induced skin barrier dysfunction, the effect of HEP on the expression of skin barrier-related proteins in TNF-α/IFN-γ-stimulated HaCaT cells was assessed using qPCR. TNF-α/IFN-γ stimulation downregulated mRNA expression levels of genes related to skin barrier formation, including *Claudin-1* and *Filaggrin* (*FLG*), compared to the untreated control group, whereas HEP treatment markedly increased both mRNA expressions in TNF-α/IFN-γ-stimulated HaCaT cells (Figure 7).

### 3.9 HEP attenuates DNFB-Induced epidermal hyperplasia and inflammatory cell infiltration in an AD-like mouse model

**FIGURE 8:**
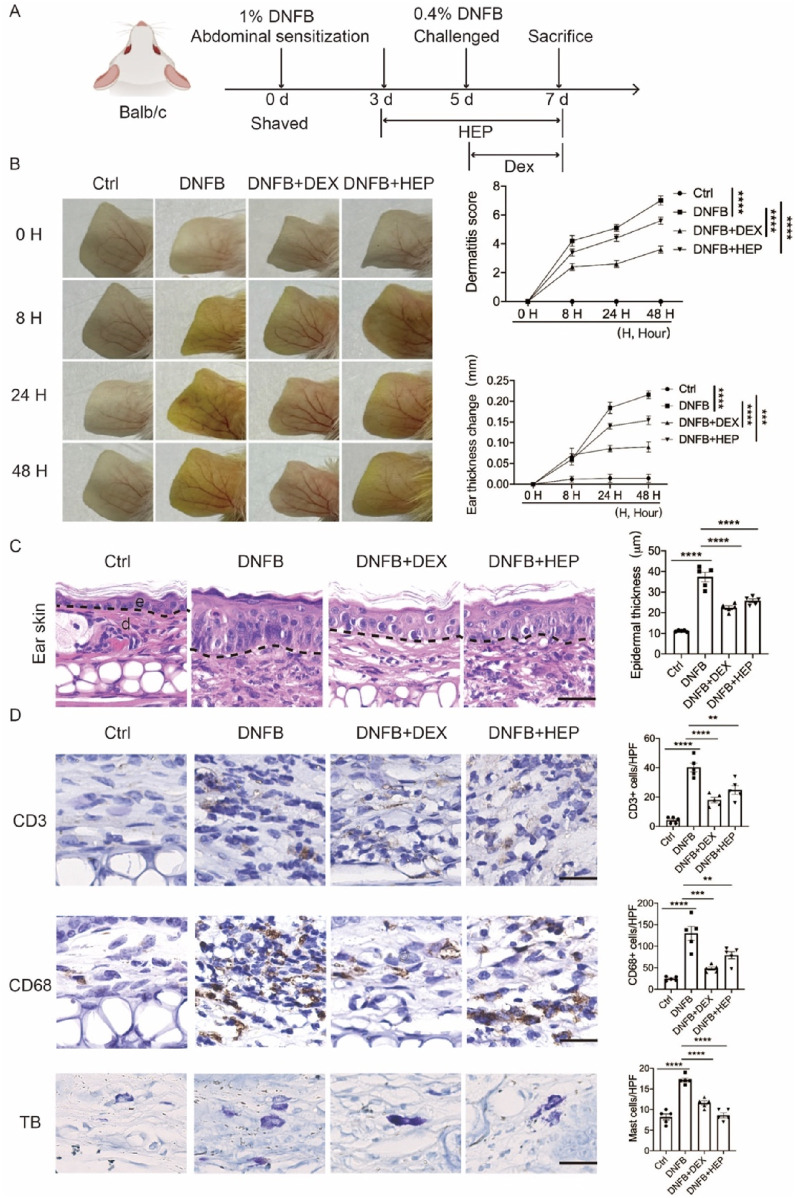
HEP ameliorates DNFB-induced skin inflammation and pathological changes in an AD-like mouse model. **(A)** Schematic representation of the experimental protocol for establishing the DNFB-induced allergic contact dermatitis model. **(B)** Representative macroscopic images showing distinct ear skin morphological changes across treatment groups: Ctrl, DNFB, DNFB + DEX, and DNFB + HEP. Quantitative analysis of ear thickness changes and clinical dermatitis scores evaluated at multiple time points in all experimental groups (n = 5). **(C)** Left, representative images of H&E stained skin sections from Ctrl, DNFB, DNFB+DEX, and DNFB+HEP mice groups (n = 5). Scale bar: 200 μm. Right, the quantitative analysis of epidermal thickness measurement. e, epidermis; d, dermis. **(D)** Left, representative immunohistochemical images showing CD3^+^ T lymphocytes, CD68^+^ macrophages, and TB-stained mast cells in dermal tissue sections from each treatment group (scale bar: 200 μm). Right, the quantitative analysis of CD3^+^, CD68^+^, and TB stained cells in skin dermal lesions. Each dot represents one mouse. Data represent the mean values ± SEM, and statistical significance was determined by two-way ANOVA for multiple comparisons. ^****^ *p* < 0.0001, ^***^ *p* < 0.001, ^**^ *p* < 0.01.

To evaluate the therapeutic potential of HEP in AD, we utilized a well-established DNFB-induced AD model that recapitulates key features of human AD (Figure 8A) (Li et al., 2013). Following DNFB challenge, characteristic inflammatory responses developed progressively, with edema and erythema manifesting at 8, 24, and 48 h post-application. Comprehensive clinical assessment using standardized dermatitis scoring revealed that both the positive control dexamethasone (DEX) and HEP treatment significantly reduced overall dermatitis severity compared to DNFB-treated controls (Figure 8B). H&E staining demonstrated that DNFB-induced lesions were characterized by pronounced epidermal hyperplasia and extensive dermal immune cell infiltration, hallmarks of inflammatory skin disease. In contrast, both DEX- and HEP-treated mice exhibited marked amelioration of these pathological changes, with substantially reduced inflammatory cell accumulation and normalization of epidermal thickness (Figure 8C).

To further characterize the inflammatory response, we performed immunohistochemical analysis targeting key immune cell populations, including macrophages (CD68), T lymphocytes (CD3), and mast cells (toluidine blue, TB). DNFB-induced skin lesions exhibited significantly increased CD68^+^ macrophages, confirming robust innate immune activation, while both DEX and HEP treatment substantially decreased CD68^+^ macrophage number, indicating the reduced macrophage infiltration and inflammatory responses. Similarly, CD3^+^ T-cell accumulation and TB-positive mast cell numbers were markedly decreased in both treatment groups compared to DNFB controls (Figure 8D). Collectively, these findings indicate that HEP exhibits potent anti-inflammatory properties *in vivo*, effectively attenuating multiple pathological features of AD-like skin inflammation through suppression of both innate and adaptive immune responses.

## 4 Discussion

The results of our study demonstrate that HEP exhibits potent antioxidant and anti-inflammatory activities in HaCaT cells. The high levels of polyphenolic and flavonoid metabolites in HEP, which are known to play a critical role in cellular defense mechanisms, indicate that HEP may serve as a promising natural therapeutic for modulating oxidative stress and inflammation. The observed modulation of key signaling pathways, including Nrf2, NF-κB, and JAK/STAT, aligns with previous studies demonstrating the involvement of these pathways in cellular antioxidant responses and immune regulation. Furthermore, the *in vivo* validation demonstrated that HEP effectively ameliorated AD-like symptoms in the DNFB-induced mouse model. These findings underscore the multifaceted role of HEP in maintaining skin homeostasis and its potential as a targeted intervention for inflammatory skin conditions such as AD.

Parsley is well-recognized for its antioxidant properties, attributed to phenolic and flavonoid metabolites such as apigenin, quercetin, and coumaric acid (Slighoua et al., 2023; Mahdi et al., 2024). Consistent with previous studies, we observed *in vitro* antioxidant activity in parsley extracts by DPPH and FRAP assays. Notably, we used three different extraction methods and found that the hydro-ethanolic extraction was most effective for obtaining high contents of polyphenol and flavonoid. Consistently, hydro-ethanolic extraction exhibited superior radical scavenging activity and ferric reducing power, which was further validated in a cell-based model where HEP mitigated t-BHP-induced oxidative stress in HaCaT cells by enhancing cell viability, reducing ROS generation, and activating antioxidant defenses.

The endogenous antioxidant system is primarily regulated by Nrf2. The activation of the Nrf2 can induce key enzymes like SOD, contributing to neutralization of ROS-mediated damaging effects by converting hydrogen peroxide into water and oxygen (De Simoni et al., 2024; Niture, Khatri, and Jaiswal, 2014; Kuai et al., 2024). Our results reveal that HEP not only scavenges ROS, but also enhances the endogenous antioxidant defenses by promoting Nrf2 nuclear translocation and upregulating SOD and CAT expression. These findings highlight the potent antioxidant capability of HEP and its therapeutic potential for protecting skin cells against oxidative damage through Nrf2 pathway activation.

Keratinocytes are central to initiating and amplifying cutaneous inflammation in AD, primarily through the release of pro-inflammatory cytokines and chemokines (Chieosilapatham et al., 2021). Among these, IL-33 plays a major role in AD initiation (Imai, 2019). Overexpressed in keratinocytes of AD patients, IL-33 is rapidly released in response to injury or infection, triggering Th2 polarization by activating immune cells such as mast cells and group 2 innate lymphoid cells (ILC2s) to produce IL-4, IL-5, and IL-13 (Schmitz et al., 2005; Salimi et al., 2013). Additionally, IL-33 promotes the secretion of pruritic cytokines, including TSLP and IL-31 from keratinocytes and Th2 cells, respectively, creating a feedback loop of scratching and cytokine release that exacerbates inflammation and barrier dysfunction (Roan, Obata-Ninomiya, and Ziegler, 2019). IL-6 and IL-8 also contribute significantly to AD pathology (Zhang et al., 2014). IL-6 facilitates Th17 differentiation, chronic inflammation, and defective skin barrier function, while elevated IL-6 levels in AD skin are linked to bacterial colonization and dermatitis severity (Zhou et al., 2007; Son et al., 2014; Kim and Kim, 2019; Navarini, French, and Hofbauer, 2011). IL-8, initially identified as a neutrophil chemoattractant, also recruits macrophages and dendritic cells in the AD microenvironment. Its levels correlate with disease severity, making it a potential therapeutic target (Matsushima, Yang, and Oppenheim, 2022; Neuber, Hilger, and König, 1991). In the present study, HEP effectively suppressed the mRNA expression of *IL-33*, *IL-6*, and *IL-8* in a dose-dependent manner in HaCaT cells stimulated by TNF-α/IFN-γ, indicating its anti-inflammatory potential.

Skin barrier defects are an initiating step in AD, allowing allergen and pathogen infiltration that triggers keratinocyte-derived cytokines, perpetuating inflammation and barrier disruption (Kim and Leung, 2018; Furue et al., 2017). Therapies targeting skin barrier have proven effective in managing AD (Schmuth et al., 2024). AD skin exhibits increased trans-epidermal water loss (TEWL), decreased stratum corneum hydration, and diminished expression of key differentiation markers, including FLG, involucrin and loricrin. FLG, a key epidermal barrier protein, contributes to maintaining skin pH and water retention (Moosbrugger-Martinz et al., 2022). Its loss, driven by genetic mutations (Palmer et al., 2006) or Th2 cytokines such as IL-4, IL-13, and IL-33, compromises the barrier, irrespective of genotype (Moosbrugger-Martinz et al., 2022; Seltmann et al., 2015; Nygaard et al., 2017; Ryu et al., 2016). Claudin-1, a tight junction protein, plays an equally crucial role by sealing intercellular spaces to prevent pathogen entry (Sumigray and Lechler, 2015). Numerous studies have demonstrated that the reduced claudin-1 expression correlates with the impaired skin barrier functions in AD (Bergmann et al., 2020; Gao, 2011). IL-33 and other Th2 cytokines can also downregulate claudin-1 expression through JAK/STAT pathway in keratinocytes (Ryu et al., 2018; Gruber et al., 2015). The present study demonstrated that HEP treatment recovered the *FLG* and *Claudin-1* expression by TNF-α/IFN-γ stimulation in HaCaT cells. These results suggest that HEP may be a useful medicinal agent for restoring the skin barrier and inhibiting chronic inflammation in AD.

The NF-κB and JAK/STAT pathway signaling pathways are central regulators of inflammatory and immune responses, and its dysregulated activation is implicated in numerous inflammatory skin disorders, including AD (Giridharan and Srinivasan, 2018; Herrington, Carmody, and Goodyear, 2016; Liu et al., 2024). Given their pivotal roles, these pathways represent promising therapeutic targets for inflammatory skin diseases. Our findings demonstrate that HEP effectively attenuates TNF-α/IFN-γ-induced inflammation in HaCaT cells by suppressing NF-κB and JAK/STAT1 pathways. This dual inhibition provides a mechanistic explanation for the anti-inflammatory effects of HEP on AD, where dysregulated NF-κB and JAK/STAT signaling drive keratinocyte-mediated inflammation and epidermal barrier dysfunction.

The *in vivo* validation using the DNFB-induced mouse AD model provided compelling evidence for the therapeutic efficacy of HEP in treating AD-like inflammation. The observed reduction in clinical dermatitis scores demonstrates the translational potential of our *in vitro* findings. Histopathological analysis revealed that HEP effectively attenuated key pathological features of AD, including epidermal hyperplasia and inflammatory cell infiltration, which directly correlate with the molecular mechanisms identified in our cell culture studies. The significant reduction in CD68^+^ macrophages, CD3^+^ T cells, and mast cells in HEP-treated mice suggests that the extract’s anti-inflammatory effects extend beyond keratinocyte-mediated responses to encompass broader immune modulation (Ganea et al., 2024). This multi-cellular therapeutic impact aligns with the observed suppression of inflammatory cytokines *in vitro* and supports the concept that HEP acts through coordinated inhibition of both innate and adaptive immune pathways. Specifically, due to the distinct pharmacological properties of DEX and HEP, HEP was intentionally administered to mouse ears 2 days prior to DEX treatment to evaluate its therapeutic potential in this study. Although DEX was more efficacious than HEP in ameliorating clinical dermatitis phenotypes and histopathological features, the well-documented adverse effects of DEX warrant further investigation of HEP as a safer alternative for long-term AD management.

The pathogenesis of AD is multifactorial, involving complex interactions between oxidative stress, inflammation, and skin barrier dysfunction. Our findings introduce the HEP as a promising anti-AD agent with triple anti-AD potency. Namely, HEP mitigated oxidative stress, suppressed inflammation and restored skin barrier integrity. These findings support its potential as a candidate for further investigation in future preclinical and clinical studies.

This study has certain limitations. First, the current research is mainly based on DNFB-induced AD mouse model, it is difficult to fully recapitulate the complex pathological dentures of human AD. Further study with alternative models, such as organoids or patient-derived xenografts, is required. Second, although we identified key signaling pathways mediated by HEP, detailed molecular mechanisms, including upstream regulators and pathway crosstalk, remain incompletely characterized. Moreover, the long-term safety and therapeutic benefits need to be further determined.

## 5 Conclusion

In the current study, HEP exhibited potent *in vitro* antioxidant activity and alleviated cellular oxidative damage by reducing intracellular ROS level and promoting antioxidant expression in human keratinocytes. HEP also effectively suppressed the inflammatory responses in keratinocytes by downregulating the expression of pro-inflammatory cytokines and upregulating the expression of skin barrier proteins. The mechanisms underlying the antioxidant and anti-inflammatory effects of HEP are associated with activating Nrf2 signaling pathway and inhibiting JAK/STAT and NF-κB signaling pathways in keratinocytes. In addition, HEP decreases the epidermis thickness and alleviates skin inflammation in DNFB-induced AD-like mouse model. These findings offer evidence supporting the potential application of HEP as a promising agent for AD therapy.

## Data Availability

The original contributions presented in the study are included in the article/Supplementary Material, further inquiries can be directed to the corresponding authors.

## Abbreviations

AD, atopic dermatitis; AP, aqueous extract of parsley; EP, Ethanolic extracts of parsley; HEP, hydro-ethanolic extract of *Parsley*; ILC2s, 2 innate lymphoid cells; JAK1/STAT1, Janus kinase 1/signal transducers and activators of transcription 1; NF-κB, nuclear factor-kappa B; Nrf2, NF-E2-related factor 2; qPCR, Quantitative real-time polymerase chain reaction; ROS, reactive oxygen species; SOD, superoxide dismutase; t-BHP, tert-Butyl hydroperoxide; TEWL, trans-epidermal water loss; DNFB, trans-epidermal water loss; DEX, Dexamethasone; TB, trans-epidermal water loss.

## References

[B1] Albanesi C. Pastore S. (2010). Pathobiology of chronic inflammatory skin diseases: interplay between keratinocytes and immune cells as a target for anti-inflammatory drugs. Curr. Drug Metab. 11 (3), 210–227. 10.2174/138920010791196328 20406192

[B2] Bahramsoltani R. Ahmadian R. Daglia M. Rahimi R. (2024). *Petroselinum crispum* (Mill.) fuss (Parsley): an updated review of the traditional uses, phytochemistry, and pharmacology. J. Agric. Food Chem. 72 (2), 956–972. 10.1021/acs.jafc.3c06429 38189231

[B3] Bellomo G. Thor H. Orrenius S. (1984). Increase in cytosolic Ca^2+^ concentration during t-butyl hydroperoxide metabolism by isolated hepatocytes involves NADPH oxidation and mobilization of intracellular Ca^2+^ stores. FEBS Lett. 168 (1), 38–42. 10.1016/0014-5793(84)80202-1 6423407

[B4] Bergmann S. von Buenau B. Vidal-y-Sy S. Haftek M. Wladykowski E. Houdek P. (2020). Claudin-1 decrease impacts epidermal barrier function in atopic dermatitis lesions dose-dependently. Sci. Rep. 10 (1), 2024. 10.1038/s41598-020-58718-9 32029783 PMC7004991

[B5] Bertino L. Guarneri F. Cannavò S. P. Casciaro M. Pioggia G. Gangemi S. (2020). Oxidative stress and atopic dermatitis. Antioxidants (Basel) 9 (3), 196. 10.3390/antiox9030196 32111015 PMC7139929

[B6] Chieosilapatham P. Kiatsurayanon C. Umehara Y. Trujillo-Paez J. V. Peng G. Yue H. (2021). Keratinocytes: innate immune cells in atopic dermatitis. Clin. Exp. Immunol. 204 (3), 296–309. 10.1111/cei.13575 33460469 PMC8119845

[B7] De Simoni E. Candelora M. Belleggia S. Rizzetto G. Molinelli E. Capodaglio I. (2024). Role of antioxidants supplementation in the treatment of atopic dermatitis: a critical narrative review. Front. Nutr. 11, 1393673. 10.3389/fnut.2024.1393673 38933878 PMC11203398

[B8] Ding X. Willenborg S. Bloch W. Wickström S. A. Wagle P. Brodesser S. (2020). Epidermal mammalian target of rapamycin complex 2 controls lipid synthesis and filaggrin processing in epidermal barrier formation. J. Allergy Clin. Immunol. 145 (1), 283–300. 10.1016/j.jaci.2019.07.033 31401286

[B9] Farzaei M. H. Abbasabadi Z. Ardekani M. R. Rahimi R. Farzaei F. (2013). Parsley: a review of ethnopharmacology, phytochemistry and biological activities. J. Tradit. Chin. Med. 33 (6), 815–826. 10.1016/s0254-6272(14)60018-2 24660617

[B10] Furue M. Chiba T. Tsuji G. Ulzii D. Kido-Nakahara M. Nakahara T. (2017). Atopic dermatitis: immune deviation, barrier dysfunction, IgE autoreactivity and new therapies. Allergol. Int. 66 (3), 398–403. 10.1016/j.alit.2016.12.002 28057434

[B11] Ganea M. Vicaş L. G. Gligor O. Sarac I. Onisan E. Nagy C. (2024). Exploring the therapeutic efficacy of parsley (*Petroselinum crispum* Mill.) as a functional food: implications in immunological tolerability, reduction of muscle cramps, and treatment of dermatitis. Molecules 29 (3), 608. 10.3390/molecules29030608 38338356 PMC10856782

[B12] Gao P. S. (2011). Reductions in Claudin-1 may enhance susceptibility to herpes simplex virus 1 infections in atopic dermatitis. J. Allergy Clin. Immunol. 128 (4), 903. 10.1016/j.jaci.2011.02.014 PMC3129463 21489616

[B13] Giridharan S. Srinivasan M. (2018). Mechanisms of NF-κB p65 and strategies for therapeutic manipulation. J. Inflamm. Res. 11, 407–419. 10.2147/jir.S140188 30464573 PMC6217131

[B14] Gruber R. Börnchen C. Rose K. Daubmann A. Volksdorf T. Madykowski E. (2015). Diverse regulation of Claudin-1 and Claudin-4 in atopic dermatitis. Am. J. Pathol. 185 (10), 2777–2789. 10.1016/j.ajpath.2015.06.021 26319240

[B15] Gulcin I. (2020). Antioxidants and antioxidant methods: an updated overview. Archives Toxicol. 94 (3), 651–715. 10.1007/s00204-020-02689-3 32180036

[B16] Heinrich M. Jalil B. Abdel-Tawab M. Echeverria J. Kulić Ž. McGaw L. J. (2022). Best practice in the chemical characterisation of extracts used in pharmacological and toxicological research-The ConPhyMP-guidelines. Front. Pharmacol. 13, 953205. 10.3389/fphar.2022.953205 36176427 PMC9514875

[B17] Herrington F. D. Carmody R. J. Goodyear C. S. (2016). Modulation of NF-κB signaling as a therapeutic target in autoimmunity. J. Biomol. Screen 21 (3), 223–242. 10.1177/1087057115617456 26597958

[B18] Imai Y. (2019). Interleukin-33 in atopic dermatitis. J. Dermatological Sci. 96 (1), 2–7. 10.1016/j.jdermsci.2019.08.006 31455506

[B19] Ji H. X. Li X. K. (2016). Oxidative stress in atopic dermatitis. Oxidative Med. Cell. Longev. 2016, 2721469. 10.1155/2016/2721469 27006746 PMC4781995

[B20] Khosravan S. Alami A. Mohammadzadeh-Moghadam H. Ramezani V. (2017). The effect of topical use of *Petroselinum crispum* (Parsley) *versus* that of hydroquinone cream on reduction of epidermal melasma: a randomized clinical trial. Holist. Nurs. Pract. 31 (1), 16–20. 10.1097/hnp.0000000000000186 27902522

[B21] Kim J. E. Kim H. S. (2019). Microbiome of the skin and gut in atopic dermatitis (AD): understanding the pathophysiology and finding novel management strategies. J. Clin. Med. 8 (4), 444. 10.3390/jcm8040444 30987008 PMC6518061

[B22] Kim B. E. Leung D. Y. M. (2018). Significance of skin barrier dysfunction in atopic dermatitis. Allergy Asthma Immunol. Res. 10 (3), 207–215. 10.4168/aair.2018.10.3.207 29676067 PMC5911439

[B23] Kuai L. Huang F. Mao L. Ru Y. Jiang J. Song J. (2024). Single-atom catalysts with isolated Cu(1)-N(4) sites for atopic dermatitis Cascade catalytic therapy *via* activating PPAR signaling. Small 20 (52), e2407365. 10.1002/smll.202407365 39363827

[B24] Kučera O. Endlicher R. Roušar T. Lotková H. Garnol T. Drahota Z. (2014). The effect of tert-butyl hydroperoxide-induced oxidative stress on lean and steatotic rat hepatocytes *in vitro* . Oxid. Med. Cell Longev. 2014, 752506. 10.1155/2014/752506 24847414 PMC4009166

[B25] Li T. Chen H. Mei X. Wei N. Cao B. (2013). Development and use a novel combined *in-vivo* and *in-vitro* assay for anti-inflammatory and immunosuppressive agents. Iran. J. Pharm. Res. 12 (3), 445–455. 24250651 PMC3813265

[B26] Li H. Zhang Z. Zhang H. Guo Y. Yao Z. (2021). Update on the pathogenesis and therapy of atopic dermatitis. Clin. Rev. Allergy Immunol. 61 (3), 324–338. 10.1007/s12016-021-08880-3 34338977

[B27] Liu S. Pi J. Zhang Q. (2022). Signal amplification in the KEAP1-NRF2-ARE antioxidant response pathway. Redox Biol. 54, 102389. 10.1016/j.redox.2022.102389 35792437 PMC9287733

[B28] Liu Z. Jiang X. Zhao K. Ruan H. Ma Y. Ma Y. (2024). Role of LECT2 in exacerbating atopic dermatitis: insight from *in vivo* and *in vitro* models *via* NF-κB signaling pathway. Front. Immunol. 15, 1439367. 10.3389/fimmu.2024.1439367 39206203 PMC11349537

[B29] Mahdi I. Imbimbo P. Annaz H. Bakrim W. B. Sahri N. Alaoui A. (2024). Profiling of *Petroselinum sativum* (mill.) fuss phytoconstituents and assessment of their biocompatibility, antioxidant, anti-aging, wound healing, and antibacterial activities. Front. Nutr. 11, 1338482. 10.3389/fnut.2024.1338482 38505264 PMC10948610

[B30] Mahmood S. Hussain S. Malik F. (2014). Critique of medicinal conspicuousness of Parsley (*Petroselinum crispum*): a culinary herb of mediterranean region. Pak J. Pharm. Sci. 27 (1), 193–202. 24374449

[B31] Matsushima K. Yang D. Oppenheim J. J. (2022). Interleukin-8: an evolving chemokine. Cytokine 153, 155828. 10.1016/j.cyto.2022.155828 35247648

[B32] Moosbrugger-Martinz V. Leprince C. Méchin M. C. Simon M. Blunder S. Gruber R. (2022). Revisiting the roles of filaggrin in atopic dermatitis. Int. J. Mol. Sci. 23 (10), 5318. 10.3390/ijms23105318 35628125 PMC9140947

[B33] Morizane S. Mukai T. Sunagawa K. Tachibana K. Kawakami Y. Ouchida M. (2023). Input/output “cytokines” in epidermal keratinocytes and the involvement in inflammatory skin diseases. Front. Immunol. 14, 1239598. 10.3389/fimmu.2023.1239598 37881433 PMC10597658

[B34] Navarini A. A. French L. E. Hofbauer G. F. (2011). Interrupting IL-6-receptor signaling improves atopic dermatitis but associates with bacterial superinfection. J. Allergy Clin. Immunol. 128 (5), 1128–1130. 10.1016/j.jaci.2011.09.009 21962991

[B35] Neuber K. Hilger R. A. König W. (1991). Interleukin-3, interleukin-8, FMLP and C5a enhance the release of leukotrienes from neutrophils of patients with atopic dermatitis. Immunology 73 (1), 83–87. 2045129 PMC1384522

[B36] Niture S. K. Khatri R. Jaiswal A. K. (2014). Regulation of Nrf2-an update. Free Radic. Biol. Med. 66, 36–44. 10.1016/j.freeradbiomed.2013.02.008 23434765 PMC3773280

[B37] Nygaard U. Van Den Bogaard E. H. Niehues H. Hvid M. Deleuran M. Johansen C. (2017). The “Alarmins” HMBG1 and IL-33 downregulate structural skin barrier proteins and impair epidermal growth. Acta Dermato-Venereologica 97 (3), 305–312. 10.2340/00015555-2552 27868148

[B38] Obmann A. Zehl M. Purevsuren S. Narantuya S. Reznicek G. Kletter C. (2011). Quantification of flavonoid glycosides in an aqueous extract from the traditional Mongolian medicinal plant Dianthus versicolor FISCH. J. Sep. Sci. 34 (3), 292–298. 10.1002/jssc.201000698 21268252

[B39] Palmer C. N. A. Irvine A. D. Terron-Kwiatkowski A. Zhao Y. W. Liao H. H. Lee S. P. (2006). Common loss-of-function variants of the epidermal barrier protein filaggrin are a major predisposing factor for atopic dermatitis. Nat. Genet. 38 (4), 441–446. 10.1038/ng1767 16550169

[B40] Pinho S. A. Oliveira P. J. Cunha-Oliveira T. (2025). Heterogeneous redox responses in NHDF cells primed to enhance mitochondrial bioenergetics. Biochim. Biophys. Acta Mol. Basis Dis. 1871 (1), 167495. 10.1016/j.bbadis.2024.167495 39241844

[B41] Pyeon S. Kim O. K. Yoon H. G. Kim S. Choi K. C. Lee Y. H. (2021). Water extract of Rubus coreanus prevents inflammatory skin diseases *in vitro* models. Plants (Basel) 10 (6), 1230. 10.3390/plants10061230 34204204 PMC8235380

[B42] Raimondo A. Serio B. Lembo S. (2023). Oxidative stress in atopic dermatitis and possible biomarkers: present and future. Indian J. Dermatol. 68 (6), 657–660. 10.4103/ijd.ijd_878_22 38371532 PMC10868981

[B43] Roan F. Obata-Ninomiya K. Ziegler S. F. (2019). Epithelial cell derived cytokines: more than just signaling the alarm. J. Clin. Investigation 129 (4), 1441–1451. 10.1172/jci124606 30932910 PMC6436879

[B44] Ryu W. I. Lee H. Bae H. C. Ryu H. J. Son S. W. (2016). IL-33 down-regulates filaggrin expression by inducing STAT3 and ERK phosphorylation in human keratinocytes. J. Dermatol. Sci. 82 (2), 131–134. 10.1016/j.jdermsci.2016.01.011 26867960

[B45] Ryu W. I. Lee H. Bae H. C. Jeon J. Ryu H. J. Kim J. (2018). IL-33 down-regulates CLDN1 expression through the ERK/STAT3 pathway in keratinocytes. J. Dermatol. Sci. 90 (3), 313–322. 10.1016/j.jdermsci.2018.02.017 29534857

[B46] Salimi M. Barlow J. L. Saunders S. P. Xue L. Z. Gutowska-Owsiak D. Wang X. W. (2013). A role for IL-25 and IL-33-driven type-2 innate lymphoid cells in atopic dermatitis. J. Exp. Med. 210 (13), 2939–2950. 10.1084/jem.20130351 24323357 PMC3865470

[B47] Schmitz J. Owyang A. Oldham E. Song Y. L. Murphy E. McClanahan T. K. (2005). IL-33, an interleukin-1-like cytokine that signals *via* the IL-1 receptor-related protein ST2 and induces T helper type 2-associated cytokines. Immunity 23 (5), 479–490. 10.1016/j.immuni.2005.09.015 16286016

[B48] Schmuth M. Eckmann S. Moosbrugger-Martinz V. Ortner-Tobider D. Blunder S. Trafoier T. (2024). Skin barrier in atopic dermatitis. J. Invest. Dermatol. 144 (5), 989–1000.e1. 10.1016/j.jid.2024.03.006 38643989

[B49] Schuler C. F. Billi A. C. Maverakis E. Tsoi L. C. Gudjonsson J. E. (2023). Novel insights into atopic dermatitis. J. Allergy Clin. Immunol. 151 (5), 1145–1154. 10.1016/j.jaci.2022.10.023 36428114 PMC10164702

[B50] Seltmann J. Roesner L. M. von Hesler F. W. Wittmann M. Weifel T. (2015). IL-33 impacts on the skin barrier by down-regulating the expression of filaggrin. J. Allergy Clin. Immunol. 135 (6):1659–1661. 10.1016/j.jaci.2015.01.048 25863977

[B51] Shi H. J. Song H. B. Gao Q. Si J. W. Zou Q. (2019). Combination of oxymatrine and diammonium glycyrrhizinate significantly mitigates mice allergic contact dermatitis induced by dinitrofluorobenzene. Exp. Biol. Med. (Maywood) 244 (13), 1111–1119. 10.1177/1535370219864895 31342769 PMC6775574

[B52] Sies H. Jones D. P. (2020). Reactive oxygen species (ROS) as pleiotropic physiological signalling agents. Nat. Rev. Mol. Cell Biol. 21 (7), 363–383. 10.1038/s41580-020-0230-3 32231263

[B53] Slighoua M. Mahdi I. Moussaid F. Z. Kamaly O. A. Amrati F. E. Conte R. (2023). LC-MS/MS and GC/MS profiling of *Petroselinum sativum* Hoffm. and its topical application on burn wound healing and related analgesic potential in rats. Metabolites 13 (2), 260. 10.3390/metabo13020260 36837879 PMC9963972

[B54] Son E. D. Kim H. J. Park T. Shin K. Bae I. H. Lim K. M. (2014). *Staphylococcus aureus* inhibits terminal differentiation of normal human keratinocytes by stimulating interleukin-6 secretion. J. Dermatol Sci. 74 (1), 64–71. 10.1016/j.jdermsci.2013.12.004 24398033

[B55] Sumigray K. D. Lechler T. (2015). “Cell adhesion in epidermal development and barrier formation,” in Cellular adhesion in development and disease. Editor Yap A. S. (San Diego: Elsevier Academic Press Inc.), 383–414. 10.1016/bs.ctdb.2014.11.027 PMC4737682 25733147

[B56] Taffe B. G. Takahashi N. Kensler T. W. Mason R. P. (1987). Generation of free radicals from organic hydroperoxide tumor promoters in isolated mouse keratinocytes. Formation of alkyl and alkoxyl radicals from tert-butyl hydroperoxide and cumene hydroperoxide. J. Biol. Chem. 262 (25), 12143–12149. 10.1016/s0021-9258(18)45328-8 2442158

[B57] Tang H. Li J. Jin M. Li C. Zhai C. Wang J. (2024). Caloric restriction impacts skin barrier function and attenuates the development of hyperplasia skin disease. Front. Nutr. 11, 1423524. 10.3389/fnut.2024.1423524 39371941 PMC11449767

[B58] Watt F. M. (2014). Mammalian skin cell biology: at the interface between laboratory and clinic. Science 346 (6212), 937–940. 10.1126/science.1253734 25414300

[B59] Woodby B. Penta K. Pecorelli A. Lila M. A. Valacchi G. (2020). Skin health from the inside out. Annu. Rev. Food Sci. Technol. 11, 235–254. 10.1146/annurev-food-032519-051722 31905017

[B60] Wullaert A. Bonnet M. C. Pasparakis M. (2011). NF-κB in the regulation of epithelial homeostasis and inflammation. Cell Res. 21 (1), 146–158. 10.1038/cr.2010.175 21151201 PMC3193399

[B61] Yang G. Seok J. K. Kang H. C. Cho Y. Y. Lee H. S. Lee J. Y. (2020). Skin barrier abnormalities and immune dysfunction in atopic dermatitis. Int. J. Mol. Sci. 21 (8), 2867. 10.3390/ijms21082867 32326002 PMC7215310

[B62] Yang N. Shao H. Deng J. Yang Y. Tang Z. Wu G. (2023). Dictamnine ameliorates chronic itch in DNFB-induced atopic dermatitis mice *via* inhibiting MrgprA3. Biochem. Pharmacol. 208, 115368. 10.1016/j.bcp.2022.115368 36493846

[B63] Zhang Z. Xiao C. Gibson A. M. Bass S. A. Khurana Hershey G. K. (2014). EGFR signaling blunts allergen-induced IL-6 production and Th17 responses in the skin and attenuates development and relapse of atopic dermatitis. J. Immunol. 192 (3), 859–866. 10.4049/jimmunol.1301062 24337738 PMC3946981

[B64] Zhou L. Ivanov I. I. Spolski R. Min R. Shenderov K. Egawa T. (2007). IL-6 programs T(H)-17 cell differentiation by promoting sequential engagement of the IL-21 and IL-23 pathways. Nat. Immunol. 8 (9), 967–974. 10.1038/ni1488 17581537

