# Supplementary material for: Petroselinum sativum (Parsley) extract suppresses oxidative stress and inflammatory responses in human keratinocytes and reduces atopic dermatitis symptoms in mouse skin
Source: Front Pharmacol. 2025 Sep 12;16:1646822. doi: 10.3389/fphar.2025.1646822 (PMC12463907; doi:10.3389/fphar.2025.1646822)
Supplement: Supplementary file 1 [file Supplementaryfile1.docx]

**Supplementary Materials**

1. *LC-MS profiling of flavonoids*

Sample analysis was conducted using an Agilent 1290 UPLC system coupled to a 6470 triple quadrupole mass spectrometer. The following reagents were used: HPLC-grade acetonitrile and methanol (ACS), formic acid (Sinopharm Chemical Reagent Co., Ltd.), and flavonoid standards (Yuanye Bio-Technology Co., Ltd.).

Approximately 0.1 g of powdered sample was placed into a 5 mL centrifuge tube. One milliliter of 70% methanol was added, and the mixture was vortexed for 1 min, ground, and then subjected to ultrasonic extraction at 4°C for 30 min. After stabilizing at 4°C for 60 min, the samples were centrifuged at 8000 rpm for 10 min. The supernatant was filtered through a 0.22 µm membrane prior to LC-MS analysis.

The flavonoids were determined using LC-MS. Briefly, chromatographic separation was performed using a Waters Acquity UPLC HSS T3 column (1.8 µm, 2.1 × 100 mm) at 40°C with a flow rate of 0.3 mL/min. The mobile phase consisted of 0.1% formic acid in water (A) and acetonitrile (B). The injection volume was 5 µL.

The flavonoid content was calculated using the following formula:

****​. W = content of target compound (mg/kg); C = concentration of target compound in sample solution (µg/L); C₀ = concentration in blank (µg/L); V = final volume (mL); N = dilution factor; m = sample weight (g). The results were shown in supplementary Table 1.

**Supplementary Table 1: The ﬂavonoid compounds in HEP extract identified by LC-MS**

| **Items** | **Contents (mg/kg)** |
| --- | --- |
| Protocatechuic acid | 62.84 |
| Protocatechuic aldehyde | 4.25 |
| Dihydrorosenin | 0.11 |
| Rutin | 256.12 |
| Vitexin | 0.21 |
| Hyperoside | 5.09 |
| Myricitrin | 4.93 |
| Taxifolin | 0.20 |
| Diosmin | 0.15 |
| Quercitrin | 0.07 |
| Astragalin | 0.84 |
| Luteolin | 1.54 |
| Quercetin | 8.55 |
| Naringenin | 0.10 |
| Apigenin | 0.32 |
| Isorhamnetin | 5.79 |
| Chrysin | 0.56 |
| Kaempferide | 0.10 |

1. *Quantification of Isorhamnetin, Quercetin and luteolin in HEP extract by HPLC*

The concentration of isorhamnetin, quercetin, and luteolin in *Petroselinum crispum* (Mill.) Fuss HEP extract was determined by high-performance liquid chromatography (HPLC) using a Zorbax SB-C18 column (4.6 × 250 mm, 5 μm; Agilent). For isorhamnetin and quercetin, the mobile phase consisted of methanol and 0.1% aqueous phosphoric acid (55:45, v/v), with a column temperature of 40 °C, detection wavelength of 370 nm, and flow rate of 1.0 mL/min. For luteolin, the mobile phase was methanol and 0.05% aqueous phosphoric acid (487:52, v/v), with a column temperature of 35 °C, detection wavelength of 360 nm, and flow rate of 1.0 mL/min. In all analyses, the injection volume was 10 μL. The extract was dissolved in 50% ethanol, sonicated, and filtered through a membrane before injection. Peaks were identified by comparing retention times and UV spectra with reference standards.

**Supplementary Table 2: The amounts of isorhamnetin, quercetin and luteolin in HEP extract.**

| **Items** | **Contents (mg/kg)** |
| --- | --- |
| Isorhamnetin | 5.1241 |
| Quercetin | 6.9745 |
| Luteolin | 1.0733 |
